# Strain-specific impacts of probiotics are a significant driver of gut microbiome development in very preterm infants

**DOI:** 10.1038/s41564-022-01213-w

**Published:** 2022-09-26

**Authors:** Lauren C. Beck, Andrea C. Masi, Gregory R. Young, Tommi Vatanen, Christopher A. Lamb, Rachel Smith, Jonathan Coxhead, Alana Butler, Benjamin J. Marsland, Nicholas D. Embleton, Janet E. Berrington, Christopher J. Stewart

**Affiliations:** 1 Translational and Clinical Research Institute, Newcastle University, Newcastle, UK; 2 Hub for Biotechnology in the Built Environment, Northumbria University, Newcastle, UK; 3 Liggins Institute, University of Auckland, Auckland, New Zealand; 4 Broad Institute of MIT and Harvard, Cambridge, MA USA; 5 Department of Gastroenterology, Newcastle upon Tyne Hospitals NHS Foundation Trust, Newcastle, UK; 6 Bioscience Institute, Newcastle University, Newcastle, UK; 7 Department of Immunology and Pathology, Central Clinical School, Monash University, Melbourne, Victoria Australia; 8 Newcastle Neonatal Service, Newcastle Hospitals NHS Trust, Newcastle, UK; 9 Population Health Sciences Institute, Newcastle University, Newcastle, UK

**Keywords:** Microbiology, Bacteriology

## Abstract

The development of the gut microbiome from birth plays important roles in short- and long-term health, but factors influencing preterm gut microbiome development are poorly understood. In the present study, we use metagenomic sequencing to analyse 1,431 longitudinal stool samples from 123 very preterm infants (<32 weeks’ gestation) who did not develop intestinal disease or sepsis over a study period of 10 years. During the study period, one cohort had no probiotic exposure whereas two cohorts were given different probiotic products: Infloran (*Bifidobacterium bifidum* and *Lactobacillus acidophilus*) or Labinic (*B. bifidum, B. longum* subsp. *infantis* and *L. acidophilus*). Mothers’ own milk, breast milk fortifier, antibiotics and probiotics were significantly associated with the gut microbiome, with probiotics being the most significant factor. Probiotics drove microbiome transition into different preterm gut community types (PGCTs), each enriched in a different *Bifidobacterium* sp. and significantly associated with increased postnatal age. Functional analyses identified stool metabolites associated with PGCTs and, in preterm-derived organoids, sterile faecal supernatants impacted intestinal, organoid monolayer, gene expression in a PGCT-specific manner. The present study identifies specific influencers of gut microbiome development in very preterm infants, some of which overlap with those impacting term infants. The results highlight the importance of strain-specific differences in probiotic products and their impact on host interactions in the preterm gut.

## Abstract (web summary)

Metagenomics and metabolomics analysis of a longitudinal cohort of 123 very preterm infants reveals multiple drivers of gut microbiome development and indicates that there are strain-specific effects of probiotic products.

## Main

Host and environmental factors shaping gut microbiome development have been well defined in term infants^1^, but less well defined in significantly preterm infants. In term infants, birth mode^2–5^ and receipt of breast milk^1,5–7^ are the main factors influencing the gut microbiome over the first year. Related work in preterm infants has yielded inconsistent results, potentially reflecting smaller cohorts and lack of longitudinal sampling^8–10^. These inconsistencies underscore the need for a focused investigation into the factors influencing normal gut microbiome structure and function in preterm infants in the absence of intestinal pathologies such as necrotizing enterocolitis (NEC) or late-onset sepsis (LOS).

Preterm infants born <32 weeks’ gestation will initially be cared for on the neonatal intensive care unit (NICU). This unique setting plays a crucial role in the acquisition and development of the gut microbiome, which has been associated with life-threatening disease including NEC^11–14^ and LOS^15,16^. This has led to increased interest in and use of probiotics in the NICU, although the efficacy of probiotics in preventing NEC and LOS remains inconclusive^17^ and the potential benefits from probiotic-mediated NEC, LOS or mortality reduction^18,19^ need to be balanced against low but important risks reported in the literature from contamination and probiotic sepsis^20–23^. Studies exploring the impact of probiotics on gut microbiome development are few in the preterm population, but have shown that *Bifidobacterium* spp. in particular are able to colonize the gut long term^24–26^.

In the present study of preterm infants in the absence of intestinal disease or LOS, we aimed to (1) characterize the longitudinal development of the preterm gut microbiome throughout their stay on the NICU and (2) determine the influence of co-variates on the developing bacterial community and function during this critical period of early life.

## Results

**Fig. 1:**
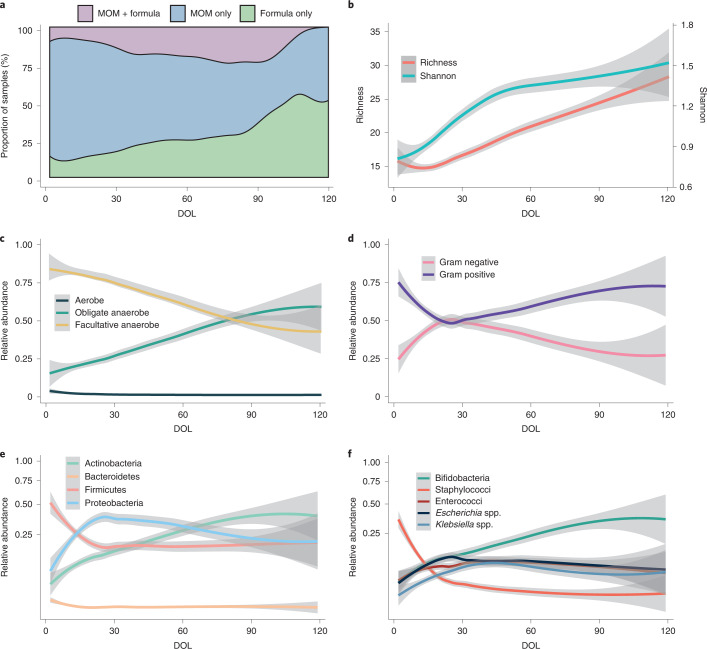
Descriptive overview of diet and the preterm gut microbiome in the first 120 d of life (*n* = 1,431). **a**, Proportion of samples where infants were receiving MOM, formula or MOM and formula. **b**–**f**, LOESS fits (95% CIs shaded in grey) over time for richness and Shannon diversity (**b**), aerobic, facultative anaerobic and obligate anaerobic bacteria (**c**), Gram-positive and Gram-negative bacteria (**d**), the top four phyla (**e**) and the top five genera (**f**).

**Extended Data Fig. 1:**
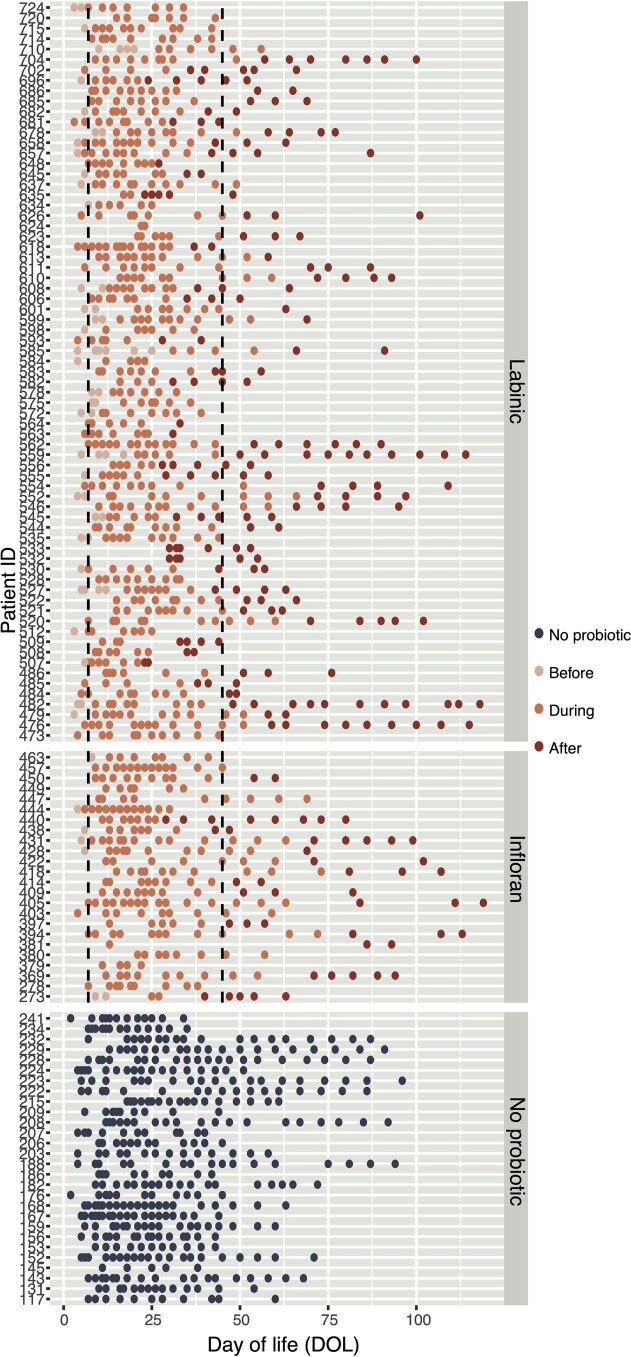
Sampling overview. Samples used in the study from birth to day 120. Dashed lines represent the overall mean start and stop day of probiotic treatment.

The current metagenomic analysis included a total of 1,431 samples collected longitudinally from 123 very preterm infants born <32 weeks’ gestation during their stay in a single British NICU (Extended Data Fig. 1). Samples were collected between birth (day of life (DOL) 0) and DOL 120, with the median (interquartile range (IQR)) DOL for final sample collection occurring on DOL 57 (43–77). Infants each contributed a median (IQR) of 11 (9–14) samples. Comprehensive demographic information is described in Methods and presented in Extended Data Table 1. Most babies received some mothers’ own milk (MOM) at some point (92.7%), with receipt of formula increasing with age. All samples had known milk exposure (MOM, formula or both) (Fig. 1a) and antibiotic exposure. To include infants from before probiotics were introduced, the cohort in the present study was admitted over a 10-year period, covering a period before probiotic introduction and during two sequentially administered probiotics. Infants born between 2011 and 2013 received no probiotics. Probiotics were then introduced to the NICU in 2013; Infloran (*B. bifidum* 1 × 10^9^ colony-forming units (c.f.u.) and *L. acidophilus* 1 × 10^9^ c.f.u.) was supplemented until mid-2016, after which Labinic (*B. bifidum* 0.67 × 10^9^ c.f.u.*, B. longum* subsp*. infantis* 0.67 × 10^9^ c.f.u. and *L. acidophilus* 0.67 × 10^9^ c.f.u.) was used.

### Overview of taxonomy

Non-bacterial microbes were explored based on Metagenomic Phylogenetic Analysis (MetaPhlAn; fungi and archaea) and VirMap (virus). No archaea and only 11 fungal species were detected. *Candida albicans* and *C. glabrata* were the most abundant and prevalent fungi, but only detected in 26 samples (14 infants) and 15 samples (9 infants), respectively. Our method allowed detection of DNA viruses, of which only two were detected; cytomegalovirus was found in eight samples from seven infants and betapolyomavirus was detected in two samples from the same infant. In total, 394 bacterial species were identified and thus subsequent analysis was focused on bacteria.

Species richness declined slightly over the first 10 d of life, corresponding to a loss of aerobic bacteria (Fig. 1b). After day 10, species richness increased consistently until NICU discharge and Shannon diversity increased exponentially from birth until day 45, with a modest increase from day 45 (Fig. 1b). There was a general increase in the relative abundance of obligate anaerobic bacteria from birth until day 80, after which the gut microbiome consisted of approximately 1:1 facultative and obligate anaerobes (Fig. 1c). Staphylococci dominated the earliest samples and accounted for most of the Gram-positive bacteria during the first month of life. Relative abundance of *Bifidobacterium* (Actinobacteria phylum) increased from birth until discharge and from day 30 was the most abundant genus. *Escherichia* and *Klebsiella*, both Gram-negative organisms from the Proteobacteria phylum, increased in relative abundance over the first month of life before gradually declining in relative abundance (Fig. 1d,e,f).

**Extended Data Fig. 2:**
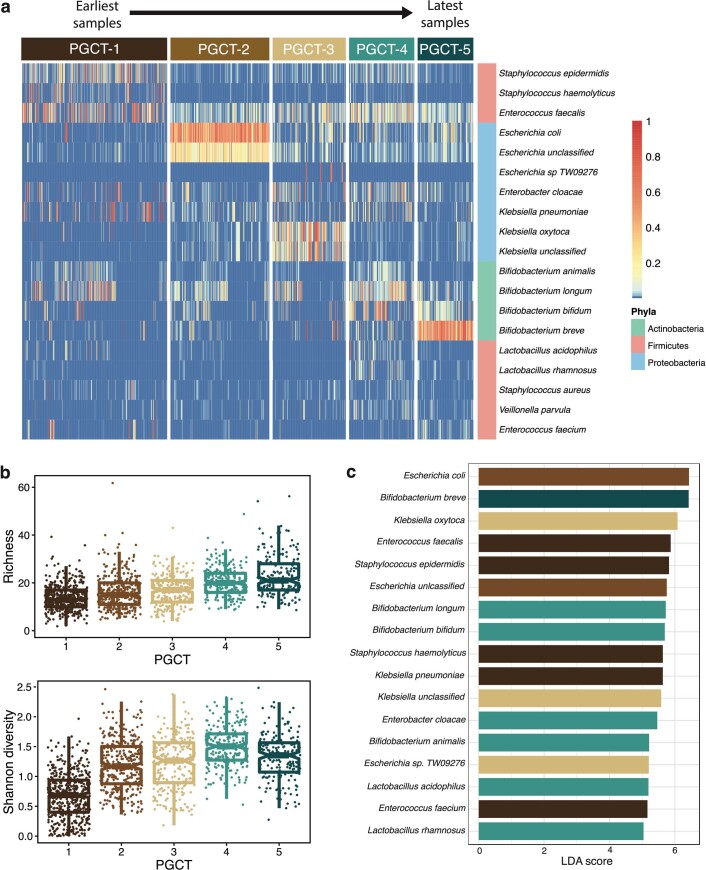
DMM clustering into PGCTs. **a**, Heatmap of all samples (*n* = 1431) showing the relative abundance of the most dominant species, coloured by phyla, stratified by PGCT. **b**, Box plots showing the alpha diversity (richness and Shannon diversity) for each PGCT. The centre line denotes the median, the box limits denote the inter-quartile range (IQR) and whiskers extend to the limits. **c**, LEfSe identifying discriminatory features of each PGCT based on Linear Discriminant Analysis (LDA). Coloured bars denote PGCTs.

Dirichlet’s multinomial mixture (DMM) modelling of bacterial species determined five clusters to be optimal, herein termed PGCTs. PGCTs were numbered 1–5 based on the average age of samples within that cluster and richness and Shannon diversity expectedly increased through each PGCT (Extended Data Fig. 2). Enterococci (*Enterococcus faecalis* and *E. faecium*) and staphylococci (*Staphylococcus epidermidis* and *S. haemolyticus*) discriminated PGCT-1; *Escherichia* spp. (*E. coli* and an unclassified species) discriminated PGCT-2; *Klebsiella* spp. (*K. oxytoca* and an unclassified species) discriminated PGCT-3; several bifidobacteria (*B. longum*, *B. bifidum* and *B. animalis*) and lactobacilli (*L. acidophilus* and *L. rhamnosus*) discriminated PGCT-4; and a single species, *B. breve*, discriminated PGCT-5 (Extended Data Fig. 2c).

### Factors shaping the preterm gut microbiome

Shannon diversity was significantly associated with DOL, probiotics (no probiotic/Infloran/Labinic), receipt of MOM (never/during/after), breast milk fortifier (BMF, never/before/during/after) and antibiotics (antibiotic in past 7 d, no/yes) (Extended Data Table 2). The direction of the effect is described further in later sections. To determine co-variates significantly associated with overall bacterial profiles, univariate permutational multivariate analysis of variance (PERMANOVA) was performed using ‘adonis’. DOL explained 4% of the total variance (effect size) in bacterial profiles (*P* < 0.001) and post-conceptional age explained 3.5% (*P* < 0.001), while unique patient identifier explained 1.8% of the variance (*P* = 0.016).

**Fig. 2:**
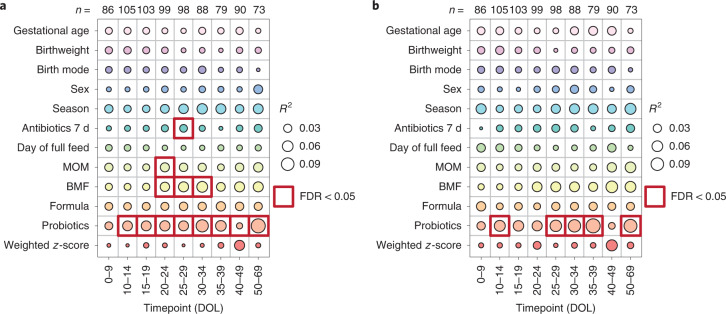
Significance and explained variance of 12 clinical co-variates at different timepoints, modelled by ‘adonis’. Bubbles show the amount of variance (*R*^2^) explained by each co-variate at a given timepoint and significant results (FDR < 0.05) are surrounded by a red box. **a**, Taxonomic profiles at the species level (*n* = 821). **b**, Functional metagenomic capacity at the enzyme level, using EC numbers (*n* = 821).

Antibiotics, MOM, BMF and probiotics were significantly associated with bacterial taxonomy at one or more timepoints (Fig. 2a). Probiotics were statistically the most significant (all *P* < 0.05) and were associated with the bacterial community at all timepoints, except days 0–9 (*P* = 0.351) which contained samples collected largely before administration began on day 7 (Extended Data Table 1). Complementary analysis on the functional metabolic capacity of the microbiome revealed only probiotics to be significantly associated, at days 10–14, 25–29, 30–34, 35–39 and 50–69 (Fig. 2b). Notably, gestational age, birthweight, birth mode, formula milk and sex were not associated with overall bacterial community composition at the taxonomic or functional level.

**Extended Data Fig. 3:**
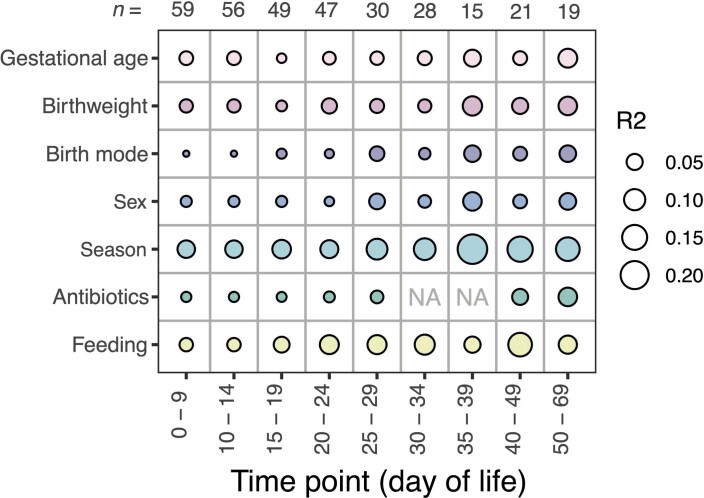
Explained variance of 7 clinical co-variates at different timepoints to validate the findings in this study, using a published metagenomic dataset from Olm *et al*^14^., modelled by ‘adonis’. Bubbles show the amount of variance (R^2^) explained by each covariate at a given timepoint. NA values are used when analyses could not be carried out, due to only 1 level of the variable existing in that given timepoint. No results were found to be significant based on taxonomic profiles at the species level.

We further sought to validate these results using a previously published metagenomic study by Olm et al.^14^ containing 86 control preterm infants (*n* = 513 stool samples), not receiving probiotics. Although feeding information was less granular than in our study, the results were generally consistent between cohorts with no significant association of any tested co-variate on the gut microbiome (Extended Data Fig. 3).

### Role of probiotics in shaping the gut community

**Fig. 3:**
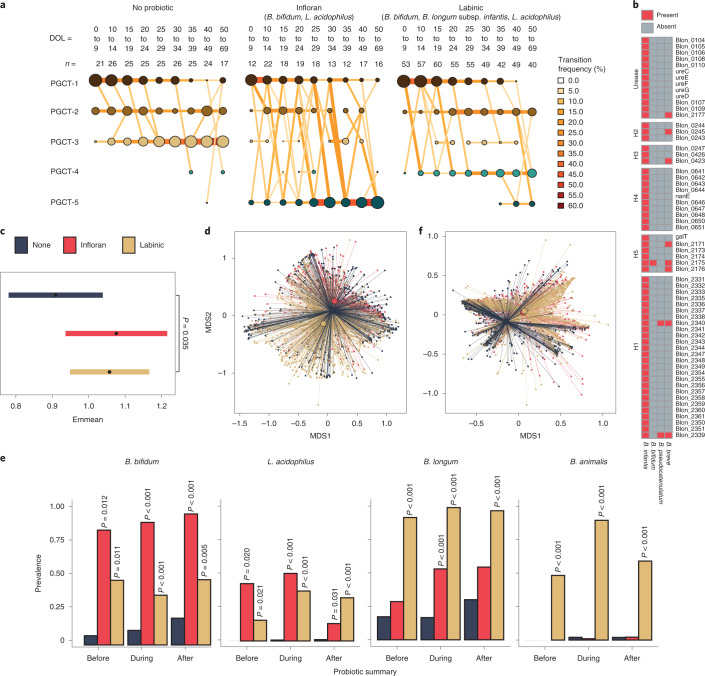
Probiotics were the most significant co-variate associated with the microbiome of preterm infants. **a**, Transition model showing the progression of samples through each PGCT from DOL 0 to DOL 69, based on probiotic type. The nodes and edges are sized based on the total counts; nodes are coloured according to PGCT and edges by the transition frequency. **b**, Prevalence of the *B. infantis* HMO gene clusters among other species. **c**, Estimated marginal means (95% CIs) representing Shannon diversity for each probiotic type, obtained from the Shannon diversity linear mixed-effects model adjusted for gestational age, birthweight, birth mode, sex, season, antibiotics, day of full feed, MOM, BMF, formula, weight *z*-scores, DOL and patient ID. The statistical significance shown is after adjustment for multiple comparisons using two-tailed Tukey’s HSD method. **d**, NMDS plot of taxonomic profiles for all samples (*n* = 1,431), showing the mean centroid for each probiotic type. **e**, Prevalence of probiotic species before, during and after probiotic treatment, stratified by probiotic type. Samples from infants who took no probiotic have been subset into three discrete time bins based on the average start and stop days for probiotic treatment (8 DOL and 44 DOL, respectively). The statistical significance shown is within probiotic summary groups (that is, before, during and after) following adjustment for multiple comparisons using Dunnett’s method, whereby samples from infants who took no probiotic were used as the control for each group. **f**, NMDS plot of EC number profiles for all samples (*n* = 1,431) showing the mean centroid for each probiotic type.

Binomial mixed-effects models showed that infants who did not receive probiotics were significantly more likely to transition into the *Klebsiella* spp.-enriched PGCT-3 (*P* = 0.021), which was also associated with a lower gestational age at birth (*P* = 0.043; Fig. 3a and Supplementary Table 1). Infants receiving Infloran were significantly more likely to transition into PGCT-5 and those receiving Labinic to PGCT-4 (both *P* < 0.001; Fig. 3a and Supplementary Table 1). Samples from PGCT-4 and PGCT-5 were from a significantly higher DOL (both *P* < 0.001; Supplementary Table 1) and thus reflected the oldest infants. PGCT-5 was dominated by *B. breve* and associated with a higher gestational age (*P* = 0.008; Supplementary Table 1). PGCT-4 was generally dominated by the species present in Labinic, including *B. longum*, *B. bifidum* and *L. acidophilus*, but also *B. animalis* (Extended Data Fig. 2c).

Multivariate Association with Linear models 2 (MaAsLin2) analysis confirmed that the relative abundance of genera (Supplementary Table 2) and species (Supplementary Table 3) present in each probiotic was significantly higher in infants receiving that probiotic. Notably, *B. breve* was significantly associated with Infloran (*P* < 0.001, *q* = 0.007) and *B. animalis* was the most significant taxa associated with Labinic (*P* < 0.001, *q* < 0.001), despite these species not being named as present in the probiotics (Supplementary Table 3). Using culture-based approaches, we were unable to culture *B. breve* from Infloran but were consistently able to culture *B. animalis* from Labinic. Given these results, we considered *B. animalis* to be present in Labinic in subsequent analyses.

Aside from probiotic species, the influence of probiotics on other naturally occurring taxa showed a significant increase in the relative abundance of *E. faecium* (*P* < 0.001, *q* = 0.004), and a significant decrease in the relative abundance of *Veillonella parvula* (*P* < 0.001, *q* = 0.022) and *Propionibacterium acnes* (*P* = 0.001, *q* = 0.030) in infants supplemented with Infloran (Supplementary Table 3). No non-probiotic species were significantly increased or decreased in infants supplemented with Labinic (all *q* > 0.05; Supplementary Table 3).

**Extended Data Fig. 4:**
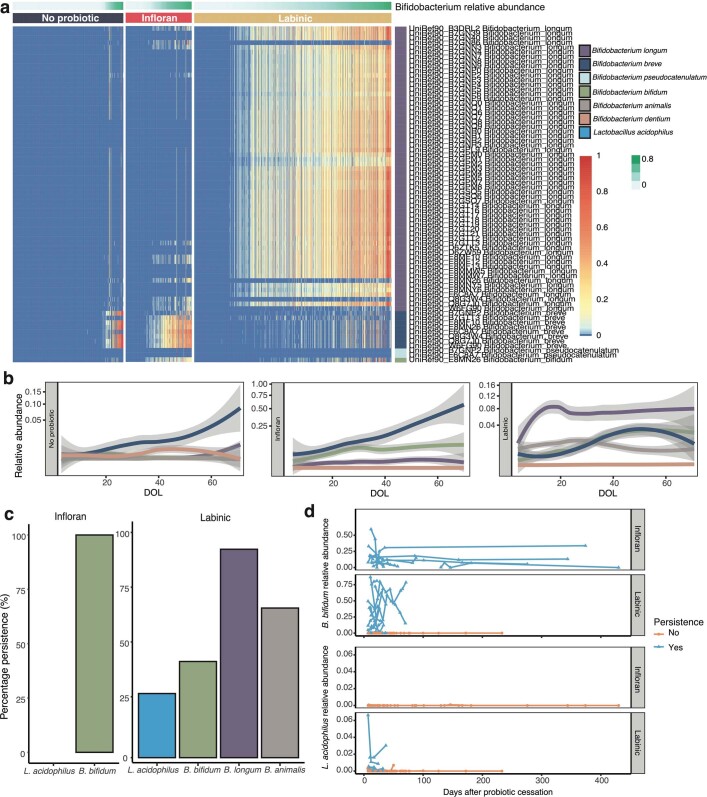
The individual impact of probiotics and probiotic species on the gut microbiome. **a**, Heatmap showing the relative abundance of *B. infantis* HMO genes and homologs in other species, coloured by species and stratified by probiotic type. **b**, Locally weighted scatterplot smoothing (LOESS) fit (95% confidence intervals shaded in grey) over time for the top 5 most dominant *Bifidobacterium* spp. **c**, Percentage persistence of probiotic species in Infloran® and Labinic™. **d**, Per-infant per-strain longitudinal abundance of probiotic species in Infloran® and Labinic™ following the cessation of probiotics.

Strain-level analyses to detect the presence of *B. longum* subsp. *infantis* was conducted using the *B. infantis* human milk oligosaccharide (HMO) gene clusters (H1, H2, H3, H4, H5 and urease), whereby samples with >90% of the genes present in those clusters were classed as having *B. infantis*^27^*. B. infantis* was detected in 672 samples, of which 666 (>99%) were from infants receiving Labinic. Additional analysis on the *B. infantis* HMO gene clusters identified homologues present in *B. breve, B. bifidum* and *B. pseudocatenulatum* (Fig. 3b and Extended Data Fig. 4a). *Bifidobacterium* spp. were also present naturally within the population, with *B. breve*, *B. dentium* and *B. longum* identified in infants who never received probiotics (Extended Data Fig. 4b).

The impact of the different probiotics is further demonstrated by significantly higher Shannon diversity in infants receiving Labinic compared with infants receiving no probiotic (*P* = 0.035) and overall microbiome profiles (Fig. 3c,d). We next sought to determine the impact on prevalence (defined as a binary yes/no) and persistence (defined as two consecutive samples where the corresponding species was detected) of the species contained within each probiotic (Methods). Compared with DOL-matched infants who never received probiotics, the probiotic species were significantly more prevalent before, during and after administration of the respective probiotic (Fig. 3e). Comparing between the probiotic groups, the prevalence of *B. bifidum* was significantly higher in Infloran compared with Labinic during (*P* < 0.001) and after (*P* < 0.001), but *L. acidophilus* prevalence was comparable before, during and after (all *P* > 0.05). Although not present in Infloran, *B. longum* was significantly more prevalent compared with DOL-matched infants who received no probiotic during Infloran treatment (*P* < 0.001), and *B. animalis* prevalence was highly specific to Labinic exposure (Fig. 3e). The analysis of persistence of *B. bifidum* and *L. acidophilus* after treatment showed further strain-specific differences between the probiotics, with no clinical co-variates other than probiotic type being significantly associated with the persistence of either species (*P* = 0.001 and *P* = 0.019, respectively). Analyses also showed increased persistence of *Bifidobacterium* spp. compared with *L. acidophilus. L. acidophilus* did not persist in any infant receiving Infloran and only in a minority of those receiving Labinic, whereas *B. bifidum* persisted in all infants receiving Infloran with non-persistence observed in infants receiving Labinic only (Extended Data Fig. 4d,e).

In accordance with taxonomic profiles, probiotics were found to be the only co-variate significantly associated with the overall functional Enzyme Commission (EC) profile at any timepoint (Fig. 2b). However, unlike taxonomic composition where probiotic groups were more dissimilar to each other than the no-probiotic group (Fig. 3d), functional profiles for infants who took either product were more similar than for infants who never took probiotics (Fig. 3f), suggesting similar functional potential regardless of which probiotic was used. MaAslin2 analysis corroborated these findings, with 346/754 (46%) significant EC numbers found to be commonly associated with both probiotic products (Supplementary Table 4). Among the significantly positively associated EC numbers, a large number of glycosylases and ligases involved in forming carbon–oxygen and carbon–nitrogen bonds were identified. In contrast, the relative abundance of numerous oxidoreductases acting on a sulfur group and other nitrogenous compounds as donors were found to be negatively associated with probiotics.

### Functional implication of PGCTs

**Fig. 4:**
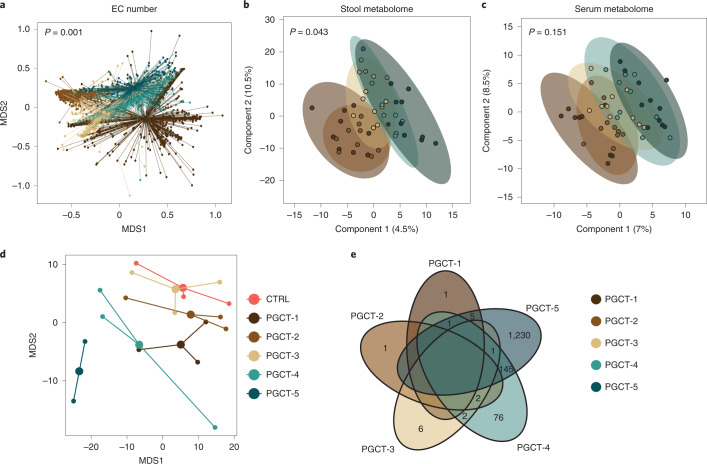
Functional implication of PGCTs. **a**, NMDS plot of EC number profiles for all samples (*n* = 1,431) showing the mean centroid for each PGCT. The statistical significance was based on PERMANOVA, with permutations constrained within the patient. **b**,**c**, PLS-DA plots of metabolite profiles (*n* = 50) showing 95% confidence ellipses for each PGCT for stool (**b**) and serum (**c**). The statistical significance was based on PERMANOVA. **d**, NMDS plot of preterm intestinal organoid transcriptome profiles (*n* = 17; 2–3 per group) showing the mean centroid for each PGCT. CTRL, control. **e**, Venn diagram showing the number of DEGs compared with control for each PGCT. Zero values were removed for clarity.

We next sought to understand whether the PGCTs, defined based on microbial taxonomy, were associated with the functional capacity of the gut microbiome. Analysis of the EC number showed that PGCTs significantly differ in their overall composition (*P* = 0.001; Fig. 4a); however, no single enzyme or pathway was found to discriminate PGCTs from each other. To further explore the functional impact of PGCTs, we selected a subset of ten stool samples representative of each PGCT (*n* = 49; one sample failed quality control (QC); Methods) and matched serum samples (*n* = 50) for untargeted metabolomics (Extended Data Table 3). Overall, stool metabolite profiles were found to significantly differ between samples based on PGCT (*P* = 0.043; Fig. 4b), whereas matched serum metabolite profiles did not (*P* = 0.151; Fig. 4c).

We next explored specific metabolites significantly enriched in PGCT-3 (associated with no probiotic infants) compared with PGCT-4/-5 (associated with probiotic infants) and vice versa (Supplementary Table 5). In stool, a single unknown metabolite was found to be significantly enriched in PGCT-3 compared with PGCT-4/-5 (*P* < 0.001, *q* = 0.0493; Supplementary Table 5). In serum, a single metabolite, lysophosphatidylcholine 20:3, was found to be significantly enriched in PGCT-4/-5 compared with PGCT-3 (*P* < 0.001, *q* = 0.01; Supplementary Table 6) and there was no metabolite correspondingly enriched in both stool and serum.

**Extended Data Fig. 5:**
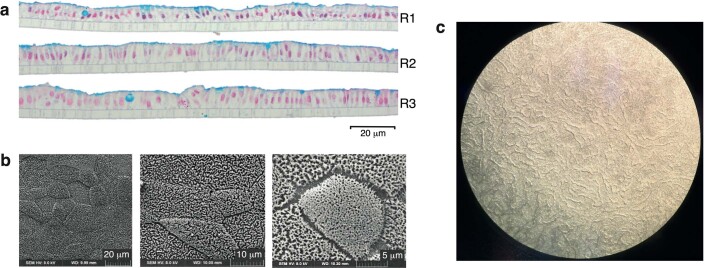
Evidence that monolayers generated from preterm intestinal-derived organoids are differentiated and entirely cover the transwell. **a**, Three technical replicates (R1, R2, R3) of alcian blue stained organoid monolayers on transwell inserts. Goblet cells and mucus layer are stained blue indicating the cells have differentiated and the goblet cells are secreting mucus apically. This further demonstrates that the monolayers are polarised and entirely cover the transwell. **b**, Scanning electron microscopy image at different magnifications showing microvilli resulting from enterocyte differentiation and that cells are contiguous (that is, no holes in monolayer). **c**, We performed regular visual inspection of the monolayers using light microscopy, and for all monolayers used in this experiment the monolayers showed full confluence across the entirety of the transwell insert.

Last, to explore the impact of small molecules from each PGCT on preterm epithelial barrier function, we employed a preterm intestinal-derived organoid model from an infant at 25 weeks’ corrected gestation under physiological oxygen conditions. The same ten stool samples from each PGCT used for metabolomics were used to create sterile faecal supernatants, before being added to differentiated intestinal organoid monolayers for 24 h (Methods). We confirmed that monolayers remained viable and confluent based on the transepithelial electrical resistance (TER) after co-culture (median 3,215.5 Ω, IQR 3170.75–3,265.5 Ω) and microscopy (Extended Data Fig. 5). Transcriptome profiles from organoids revealed a specific host response to each PGCT faecal supernatant, with PGCT-4 and PGCT-5 clustering distinctly from the other conditions on the *x* axis (Fig. 4d). This is further supported by PGCT-4- and PGCT-5-exposed organoid monolayers showing the most differentially expressed genes (DEGs) compared with the media controls (Fig. 4e). Due to insufficient DEGs being identified for other PGCTs versus control, gene ontology (GO) and enrichment analysis were carried out for PGCT-4- and PGCT-5-exposed monolayers only. Grouping genes upregulated in PGCT-4- and PGCT-5-exposed monolayers by GO revealed various biological processes to be enriched, with a number of cellular and metabolic processes, including cellular protein metabolic processes (Supplementary Table 7).

### Modulation of the infant microbiome by diet and antibiotics

**Fig. 5:**
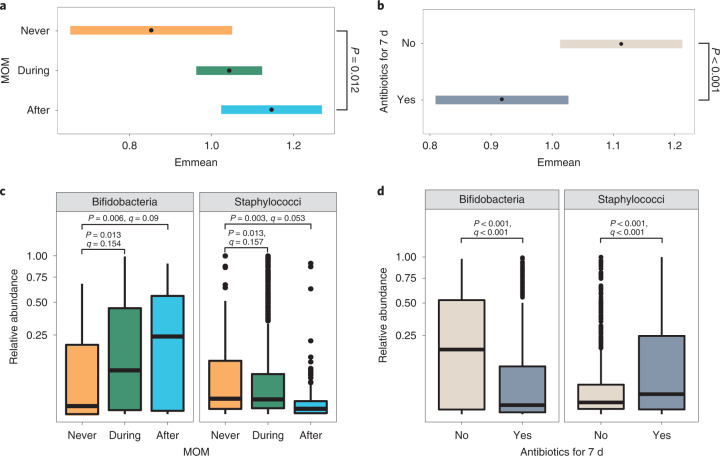
MOM and antibiotics are significantly associated with the preterm gut microbiome. **a**,**b**, Estimated marginal means (95% CIs) representing Shannon diversity for MOM (**a**) and antibiotics (**b**), obtained from the Shannon diversity, linear, mixed-effects models adjusted for gestational age, birthweight, birth mode, sex, season, day of full feed, BMF, formula, probiotics, weight *z*-scores, DOL and patient ID. The statistical significance shown is after adjustment for multiple comparisons using the two-tailed Dunnett’s method, whereby ‘never’ or ‘no’ was used as the control, respectively. **c**,**d**, Box plots showing the relative abundance of bifidobacteria and staphylococci in all samples (*n* = 1.431) across MOM (**c**) and antibiotic (**d**) groups. The centre lines denote the median, the box limits denote the IQR and the whiskers extend to the limits. Points outside the whiskers represent outliers. Statistical significance is based on *P* values and *q* values obtained from MaAsLin2 analysis.

Receipt of BMF, MOM and antibiotics was significantly associated with Shannon diversity (Extended Data Table 2) and the gut microbiome profiles around 1 month of life only (Fig. 2). Shannon diversity was significantly higher after receipt of MOM compared with never receiving MOM (*P* = 0.012; Fig. 5a) and was significantly reduced in samples where antibiotics had been given in the previous 7 d (*P* < 0.001; Fig. 5b).

Samples collected during BMF were more likely to belong to *Escherichia* spp.-dominant PGCT-2 (Supplementary Table 1), have higher *Escherichia* genus (Supplementary Table 2) and an unclassified *Escherichia* sp. (Supplementary Table 3). Compared with infants who never received MOM, the relative abundance of bifidobacteria was significantly higher in samples collected during (*P* = 0.013, *q* = 0.154) and after (*P* = 0.006, *q* = 0.09) receipt of MOM, and the relative abundance of staphylococci was significantly lower during (*P* = 0.013, *q* = 0.157) and after (*P* = 0.003, *q* = 0.053; Fig. 5c and Supplementary Table 2). Analysis at the species level did not find specific *Bifidobacterium* spp. to be significantly enriched with MOM, whereas lower staphylococci were primarily driven by *S. aureus* (Supplementary Table 3). Inverse associations were observed in infants who received antibiotics, where receipt of antibiotics in the previous 7 d significantly reduced the relative abundance of bifidobacteria (*P* < 0.001, *q* < 0.001) and increased staphylococci (*P* < 0.001, *q* < 0.001; Fig. 5d and Supplementary Table 2). At the species level, *B. bifidum*, *B. longum* and *B. breve* were significantly reduced (all *P* < 0.01, *q* < 0.1) and *S. haemolyticus*, *S. warneri* and *S. lugdunensis* were significantly increased (all *P* < 0.01, *q* < 0.2; Supplementary Table 3).

At a functional level, the relative abundance of a single enzyme, a transaldolase (EC 2.2.1.2), was found to be the most significantly associated EC number both during and after receipt of MOM (Supplementary Table 4). The relative abundance of various other transferases was also found to be positively associated during and after receipt of MOM, particularly glycosyltransferases and those involved in the transfer of one-carbon- and phosphorus-containing groups. In contrast, the relative abundance of most of the EC numbers was negatively associated with receipt of antibiotics in the past 7 d, such as enzymes involved in forming carbon–nitrogen bonds and transfer of one-carbon groups (Supplementary Table 4). EC numbers positively associated with antibiotics include oxidoreductases acting on sulfur groups and those acting on paired donors.

## Discussion

We present the largest longitudinal metagenomic analysis of very preterm infants who did not develop intestinal complications or sepsis. Where administered, probiotics were the primary factor influencing the preterm gut microbiome, followed by receipt of antibiotics, MOM and BMF. Two different probiotic products altered the transition of the microbiome into different PGCTs, both characterized by samples collected at the oldest postnatal ages. The PGCTs were enriched in different *Bifidobacterium* spp. and showed differences in their functional implications and interaction with the host epithelium.

Other findings validated in the Olm et al.^14^ cohort highlight important differences in comparison to term infants^2,28,29^. Birth mode was not associated with the microbiome and the total variance explained by co-variates was around tenfold lower than observed in term infants^1^. This suggests that the NICU practices and environment dominate the preterm microbiome, which is important when interpreting findings from different settings.

Over this 10-year observational study, we were able to investigate the impact of two different probiotic products that were used during discrete time periods and before probiotics were ever used. Once a probiotic was in use, probiotic species were detected in stool before deliberate administration. This ‘unit cross-contamination’ has been seen in previous studies^30–32^ and has important implications for probiotic trial design.

Previous studies in preterm infants have shown probiotics to alter the gut microbiome^24–26,33,34^. In the present study, the probiotic product was identified as the main driver in shaping the bacterial community at both a taxonomic and a functional level. We showed that supplementation of either Infloran or Labinic was associated with transition into two different *Bifidobacterium* spp.-enriched PGCTs (PGCT-4 and -5), both of which reflected samples obtained from the oldest infants. Previous studies have found *Bifidobacterium* spp.-enriched PGCTs to be associated with positive health outcomes^13^, but the functional implications of this have not previously been explored.

To determine the relevance of PGCTs on host–microbe interaction, we performed metabolomics on matched stool and serum samples, and used an experimental preterm intestinal organoid model. Overall, metabolite profiles of stool, but not serum, were associated with the PGCT. In addition, sterile faecal supernatants containing the metabolites and other components of stool were found to impact preterm epithelial response in a PGCT-specific manner. Of note, although a healthy section of tissue was used for organoid generation, intestinal organoid models derived from preterm infants require a patient to have a clinical complication needing surgery (in this case NEC) and so are not healthy individuals. The intestinal region (that is, small or large intestine) and maturity of the patient may also impact host transcription^35^. Although further work is needed to determine the potential biological significance of the functional changes resulting from probiotic administration, this demonstrates that transition into different PGCTs, driven by probiotic use, has associated functional implications.

It is important to note that excretion of supplemented strains in stool collected during treatment does not necessarily imply intestinal colonization. We therefore included assessment of the persistence of strains after stopping probiotics. Several studies have shown individual differences in probiotic and transient microbe colonization^36–38^, as well as differences in the persistence of probiotic species after treatment, particularly higher persistence of bifidobacterial strains compared with lactobacilli^24–26^. We also observed individualized differences in probiotic colonization. All *Bifidobacterium* spp. showed higher persistence compared with *L. acidophilus* and the persistence of *B. bifidum* and *L. acidophilus* (that is, the two strains present in both probiotics) was dependent on the probiotic used. The lower persistence of *L. acidophilus* may reflect the preterm gut ecosystem not being optimal for this species, because it is not a commonly abundant or persistent member of the preterm gut microbiome^24,26,33,39^. These results highlight altered short- and long-term colonization depending on the probiotics/strains used, emphasizing the importance of better understanding of short- and long-term impacts at the strain level.

Despite the apparent importance of probiotics in this population in providing an early source of *Bifidobacterium* spp., we also identified natural *Bifidobacterium* colonizers, namely *B. breve, B. dentium* and *B. longum* subsp. *longum*. It has been widely reported that MOM has a bifidogenic effect through the provision of HMOs^40–43^. All *Bifidobacterium* spp. detected in this preterm cohort have been previously shown to utilize HMOs for growth, with notable variation at the strain level^40^. Notably, we identified MOM to be associated with an increased relative abundance of bifidobacteria and decreased relative abundance of staphylococci, whereas antibiotics were associated with a decreased relative abundance of bifidobacteria and increased relative abundance of staphylococci.

In summary, we show, in a large and extensively longitudinally sampled population of preterm infants, that the choice of probiotic product impacted development of the gut microbiome in different ways, accelerating transition into *Bifidobacterium* spp. *-*dominant PGCT-4 or -5, which reflected bacterial communities of the oldest samples. In addition, these PGCTs showed differences in their functional implications and interaction with the host epithelium. These results help provide a framework and identify important aspects for consideration when designing interventional trials targeting the gut microbiome of preterm infants.

## Methods

### Cohort and study design

The present study included 123 preterm infants born at <32 weeks’ gestation without congenital anomaly, early onset sepsis, LOS, NEC, focal intestinal perforation or other intestinal pathology. These morbidities were excluded because they are most strongly associated with changes in the gut microbiome. No statistical methods were used to predetermine sample size, but our sample size is larger than those reported in previous publications. As an observational, retrospective study, no subject randomization was required. Data collection and analysis were not performed blind to the conditions of the experiments.

Infants were recruited to the Supporting Enhanced Research in Vulnerable Infants Study (SERVIS) with written parental consent covering data and sample collection. The study protocol was approved by Newcastle Hospitals NHS Foundation Trust, NRES Committee North East and N. Tyneside 2 10/H0908/39, and the research complies with all relevant ethical regulations. All work with clinical samples, including organoids, is covered within these ethical approvals.

All infants were cared for in the NICU of the Royal Victoria Infirmary, Newcastle, with standardized feeding and antibiotic and antifungal guidelines (prophylactic fluconazole). The earliest included infants were born in 2011 and probiotics were introduced into routine use in 2013. Between 2013 and 2016, infants received the probiotic Infloran (*B. bifidum* 1 × 10^9^ c.f.u. and *L. acidophilus* 1 × 10^9^ c.f.u.); then, due to lack of availability, after mid-2016 Labinic (*B. bifidum* 0.67 × 10^9^ c.f.u.*, B. longum* subsp*. infantis* 0.67 × 10^9^ c.f.u. and *L. acidophilus* 0.67 × 10^9^ c.f.u.) was used. Stool samples used in the analysis were collected longitudinally (*n* = 1,431) from day 0 until day 120, alongside extensive clinical metadata for each infant, including demographics and treatments such as feed exposures.

Variables that are fixed through time (for example, gestational age, birth mode, sex) are described on a per-infant basis and are thus constant for all samples from a given infant. Other variables were categorized to reflect exposure in relation to time (for example, antibiotics, receipt of MOM), and therefore are on a per-sample basis. Specifically, the clinical variables used were gestational age at birth (continuous; range 23–31), birthweight (continuous; range 500–2,000 g), birth mode (vaginal/caesarean), sex (male/female), season at birth (winter/spring/summer/autumn), intravenous antibiotics in the past 7 d (no/yes), day of full feed (continuous; range 6–39), MOM (never/during/after), BMF (never/before/during/after), formula (never/before/during/after), probiotics (no probiotic/Infloran/Labinic) and weight *z*-score difference between birth and discharge (continuous; range −5.4 to 1.1).

For the persistence analysis (described in more detail in Statistical analysis), all co-variates needed to be on a per-infant basis, restricting this analysis to gestational age, birthweight, birth mode, sex, season, total number of antibiotic courses, day of full feed, BMF ever (no/yes), formula ever (no/yes), probiotics and weight *z*-score change. MOM could not be included in this particular analysis because there was only one baby who did not receive MOM in this subset.

### Metagenomic shotgun sequencing, taxonomic and functional profiling

DNA was extracted from ~0.1 g of stool using the DNeasy PowerSoil Kit (QIAGEN) following the manufacturer’s protocol, and sequencing was performed on the HiSeq X Ten (Illumina) with a read length of 150-bp paired-end reads. Taxonomic profiling of metagenomic samples was performed using MetaPhlAn v.2.0 (ref. ^44^) (bacterial, archaeal and fungal taxonomic classification) and VirMAP v.1.0 (ref. ^45^) (viral taxonomic classification) based on default settings. Functional profiling was performed using HUMAnN v.2.0 (ref. ^46^) based on default settings. Microbial enzymes (level-4 EC categories) were quantified by summing the abundances of individual gene families mapping to each EC number based on UniRef90-EC mapping from UniProt^47^.

### Untargeted metabolomics on stool and serum samples

A subset of ten stool samples representative of each PGCT and matched serum were selected for untargeted liquid chromatography–mass spectrometry (LC–MS). As PGCTs were strongly associated with DOL at sampling, samples were primarily chosen to match for DOL between PGCTs to mitigate confounding by age at sampling. Other clinical variables were matched in addition, including gestational age, birthweight, birth mode and sex. Based on these criteria, no clinical variable was significantly different between PGCTs (all *P* > 0.05; Supplementary Table 5). Metabolites from these samples were extracted using a methanol solvent solution, supplemented with 1 µM MS internal standards (CAPS, CHAPS and PIPES) and 5 µM 2,6-di-*tert*-butyl-4-methylphenol. Serum samples were centrifuged at 800*g* for 5 min, supernatants were collected and the solvent solution was added at a 4:1 ratio. Samples were shaken for 1 h at 4 °C, centrifuged at 14,000*g* for 10 min and supernatants collected. Liquid from stool samples was evaporated using a Speedvac (Thermo Fisher Scientific) and a solvent solution was added at a ratio of 300 µl per 10 µg. Samples were shaken for 1 h at 4 °C, followed by centrifugation at 14,000*g* for 20 min, and supernatants were collected.

The LC–MS data were acquired on a Dionex Ultimate 3000 RS high-performance liquid chromatography system (Thermo Fisher Scientific) coupled with a Q-Exactive Orbitrap mass spectrometer (Thermo Fisher Scientific). Chromatographic separation was performed on a ZIC-pHILIC column (5 µm, polymeric, 150 × 4.6 mm^2^, SeQuant, Merck). Mobile phase (A) was 20 mM ammonium carbonate and (B) acetonitrile. The gradient programme started at 80% (B) and reduced to 50% (B) over 15 min, then reduced to 5% (B) over 3 min, where washing occurred for 3 min; finally there was an 80% (B) re-equilibration for 8 min. The flow rate was 0.3 ml min^−1^ and the column compartment temperature was 40 °C. Total run time was 32 min with an injection sample volume of 10 µl. The mass spectrometer operated in positive and negative polarity, switching at 35,000 resolution and 200 *m*/*z* with detection range of 85–1,275 *m*/*z* in full-scan mode. An electrospray ionization source (HESI) was set to 3.5 kV voltage for positive mode and 4.0 kV for negative mode, sheath gas was set to 50 and aux gas to 20 arbitrary units, capillary temperature 300 °C and probe heater temperature 120 °C. Serum samples were analysed as a single batch, as were stool samples. Each sample set was randomized to account for system drift. Mixtures of pure authentic standards containing approximately 320 metabolites were acquired as separate injections and used to confirm retention times.

The raw LC–MS data of both serum and stool samples were independently processed as stated in the metabolome–lipidome–MS-DIAL pipeline (Code availability) using MS-DIAL v.4.8 (ref. ^48^). Metabolomic processing was conducted in positive and negative ion mode. Default parameters were applied unless otherwise stated. Peak detection parameters included a minimum peak amplitude of 100,000. Peaks were identified using the MassBank database v.2021.02 (ref. ^49^) with a retention time tolerance (RTT) of 0.1 min, accurate mass tolerance (AMT) of 0.002 Da and identification score cut-off of 80%. Peaks were aligned using an RTT of 0.3 min and AMT of 0.002 Da, with gap filling by compulsion. MS/MS was exported and further processed for secondary annotation using the Global Natural Products Social Molecular Networking feature-based molecular networking tool^50^.

Peak intensity tables were exported from MS-DIAL and the R package pmp v.1.6.0 (ref. ^51^) was used for the following QC and pre-processing steps. Peaks were filtered for intensities at least fivefold higher than LC–MS blanks, samples with >80% missing values, features with >20% missing values and peaks filtered based on the percentage of variation in the QC samples with a maximum relative s.d. of 25%. Based on this, one stool sample from PGCT-2 was excluded. Data were normalized using probabilistic quotient normalization, followed by Random Forest missing data imputation using the missForest R package v.1.4 (ref. ^52^) and subsequent generalized logarithmic (glog) transformation. MS1 data were further annotated using the human metabolome database (HMDB, v.4, July 2021)^53^, with an AMT of 0.002 Da. Any unannotated features were removed. The remaining dataset was subject to manual feature curation in MS-DIAL, where poor quality spectral features were removed.

### Preterm intestinal organoid co-culture

A human intestinal organoid line was generated from preterm intestinal ileum tissue after surgical resection for NEC^54^. The infant was a boy born at 24 weeks’ gestation and had surgery on DOL 10.

Intestinal organoids (*n* = 3 technical replicates) were exposed to pooled faecal supernatants representing each PGCT and a control containing no faecal supernatant. Sterile faecal supernatants were prepared using a modified method described elsewhere^55^. Briefly, ~0.25 g of stool (*n* = 10) was pooled for each PGCT and diluted in 25% (w/v) sterile phosphate-buffered saline before being vortexed for 20 min with glass beads. Faecal slurries were centrifuged for 20 min at 1,600*g* and 4 °C, the supernatant was re-centrifuged for 10 min at 14,000*g* and 4 °C, and the resulting supernatant was serially filtered (0.45 µm and 0.22 µm). Faecal supernatant was stored at −80 °C until use.

Intestinal organoids were seeded as monolayers on 0.4 µm Transwells (Corning) and, after reaching confluence (~2 d), were differentiated for 4 d^56^.

Co-culture of preterm intestinal organoid monolayers with sterile faecal supernatants was performed for 24 h using the organoid anaerobe co-culture (OACC) model^57^. The sterile faecal supernatants were added apically, corresponding to the intestinal lumen. The OACC model was used to recapitulate the steep oxygen gradient across the epithelium and mimic the low oxygen gradient of the ileum. TER was measured at the end of the experiment to confirm that all monolayers remained intact and cells were contiguous.

### RNA-seq

After 24 h of exposure, RNA was extracted from organoid monolayers using the RNeasy kit (QIAGEN) before undergoing RNA-sequencing (RNA-seq) at the Newcastle University Genomics Core Facility. One sample from the PGCT-5 exposure failed QC and was not included in the subsequent analysis. Briefly, stranded messenger RNA-seq libraries were prepared using the TruSeq Stranded mRNA kit (Illumina) and IDT for Illumina TruSeq RNA UD Index adapters following the manufacturer’s protocol. Libraries were quantified using a TapeStation 4200 (Agilent Technologies) and Qubit 4 (Thermo Fisher Scientific) and equimolar pooled. The pooled library was sequenced at ~50 million 100 bp single-reads per sample on a NovaSeq 6000 using an S2 100 cycle flow cell (Illumina). Data for individual samples were demultiplexed into separate FASTQ files using Illumina’s bcl2fastq software.

QC of raw reads was performed using fastq_quality_trimmer from the FASTX Toolkit v.0.0.14 before being mapped to the human transcriptome (GRCh38.p13) using Salmon v.0.13.1 (ref. ^58^) to estimate transcript abundance. Estimated count data were aggregated at the gene level by tximport^59^ for downstream analysis. DESeq2 v.1.32.0 (ref. ^60^) was used to normalize RNA-seq count data and identify DEGs between PGCT and control replicates. Genes were considered differentially expressed if they displayed an absolute positive or negative fold-change of ≥1.5 and a false discovery rate (FDR)-adjusted *P* < 0.05. A Venn diagram of DEGs was produced using the VennDiagram package v.1.7.1 (https://cran.r-project.org/web/packages/VennDiagram/index.html).

### Statistical analysis

All statistical analyses were performed in R v.4.0.2 (https://www.r-project.org). Unless stated otherwise, all visualizations were plotted using the ggplot2 package v.3.3.2 (ref. ^61^). Where necessary, data were formally tested for normality and equal variances. Shannon diversity and richness were calculated for each sample using the vegan package v.2.5-7 (https://cran.r-project.org/web/packages/vegan/index.html). Both Shannon diversity and relative abundance data were modelled using LOESS (locally weighted estimated scatterplot smoothing) regression and plotted with 95% confidence intervals (CIs). All permutation tests (that is, PERMANOVA) were conducted with 10,000 permutations.

### Determining PGCTs

DMM was used to cluster samples on the basis of microbial community structure^62^ and to determine the PGCTs for all samples. Five PGCTs were found to be optimal on the basis of the lowest Laplace approximation score. PGCTs were manually ordered from the youngest (PGCT-1) to the oldest (PGCT-5), based on the average DOL of samples within each PGCT.

The linear discriminant analysis effect size method^63^ was used to determine the bacterial species and EC numbers that discriminated each cluster using MicrobiomeAnalyst^64,65^.

### PERMANOVA

To determine which co-variates were associated with metagenome profiles while accounting for repeated measures, multiple cross-sectional analyses using the ‘adonis’ function from the vegan package were performed. Data were split into nine specific time windows based on DOL, which were chosen to both maximize the number of samples within each window and reflect the progression of enteral feed independence as follows: establishing enteral feeds (0–9), reaching full feeds (10–14), independent of parenteral nutrition (15–19) and maturation on full enteral feeds (20–24, 25–29, 30–34, 35–39, 40–49, 50–69). For the univariate analysis, too few infants/samples were available beyond this timepoint. Only a single sample per infant, the earliest available, was included within each time window. The association of 12 clinical variables (defined in the cohort section) on the metagenome and functional profiles was tested, based on Bray–Curtis dissimilarity. Each test was performed in a stepwise manner and subsequent *P* values were adjusted for multiple comparisons using FDR adjustment (Benjamini–Hochberg procedure^66^).

To assess whether there was a statistically significant difference in serum and stool metabolite profiles based on PGCT assignment, PERMANOVA was performed in MetaboAnalyst v.5.0 (ref. ^67^).

### Ordination

For metagenomic and RNA-seq analysis, ordinations were performed on all data using non-metric multidimensional scaling (NMDS). NMDS plots were based on Bray–Curtis dissimilarity matrices for both taxonomic and functional data and Euclidean distance on regularized logarithm (rlog), transformed, normalized RNA-seq count data, using the ‘metaMDS’ function from the vegan package. The mean centroid for each group was calculated and plotted.

For metabolite analysis of stool and serum samples, ordinations were performed on all data using partial least-squares discriminant analysis using MetaboAnalyst v.5.0 (ref. ^67^).

### LMMs and GLMMs

Various linear mixed models (LMMs) and generalized LMMs (GLMMs) were fit to the data using the glmmTMB package v.1.0.2.1 (ref. ^68^) or, alternatively, the logistf package v.1.24 (https://cran.r-project.org/web/packages/logistf/index.html), which was used to fit logistic regressions using Firth’s bias-reduced penalized likelihood, when there was quasi-complete or complete separation. To detect separation and infinite maximum likelihood estimates in binomial logistic regression models, the ‘detect_separation’ and ‘check_infinite_estimates’ functions from the brglm2 package v.0.7.1 (https://cran.r-project.org/web/packages/brglm2/index.html) were used. Model validity was assessed using diagnostic residual plots, generated by the DHARMa package v.0.3.3.0 (https://cran.r-project.org/web/packages/DHARMa/index.html). Diagnostic residual plots were not generated for models fit by logistf. The general formula for each of the LMMs fitted was as follows:

$$Y\approx X_1 + X_2 + \ldots + X_n + (1|{\mathrm{Subject ID}}).$$

To find out which co-variates were significantly associated with Shannon diversity, mixed-effects models were fit using the Gaussian distribution. All 12 co-variates included in the ‘adonis’ analysis plus DOL were included as fixed effects and subject ID was included as a random group intercept.

To determine which co-variates were significantly associated with the five PGCTs, individual mixed-effects binomial logistic regression models were fit, one for each cluster versus all other clusters. Each model contained the same 12 co-variates plus DOL as fixed effects and subject ID as a random group intercept. Mixed-effects binomial logistic regression models were also fit to assess the prevalence of probiotic species. DOL had an effect on the relative abundance of probiotic species, so the before, during and after probiotic groups are nested in time. To account for this, the control group of samples from infants who had taken no probiotic was subset into three distinct time bins. These specific time bins were based on the mean start DOL for probiotics (8 DOL) and the mean stop DOL for probiotics (44 DOL). Mixed-effects binomial logistic regressions were fit separately within groups (before, during and after probiotics) for each probiotic species. They were also fit separately between groups (Infloran and Labinic) for each species.

Where used, global *P* values for fixed effects from the final models were obtained by analysis of variance (type II Wald’s χ^2^ test) from the car package^69^ v.3.0-10. All post-hoc analysis was performed using either pairwise comparisons (Tukey’s highly significant difference (HSD) method) or treatment versus control comparisons (Dunnett’s test), both adjusting for multiple comparisons, using the emmeans package v.1.5.4 (https://cran.r-project.org/web/packages/emmeans/index.html).

### Analysis of probiotic ‘persisters’ to determine significant co-variates

Persistence analysis included all infants receiving probiotics that had at least two samples at least 7 d after probiotics were stopped, and included an additional 22 samples taken after 120 DOL. Persistence of *B. bifidum* and *L. acidophilus* was assessed, because these two species were found in both Infloran and Labinic, allowing for a larger sample size (*n* = 52). Infants were classed as ‘non-persister’ for a species if there were two consecutive samples with a relative abundance of 0. This criterion was found to be optimal and babies could be separated quite clearly into ‘persisters’ and ‘non-persisters’. Binomial logistic regressions were fit for each probiotic species to determine which co-variates were significantly associated with persistence, using the logistf package as previously described. The models both included the subject-level co-variates as described.

### MaAsLin analysis to determine significant taxa and EC numbers associated with each co-variate

The MaAsLin2 package v.1.2.0 (ref. ^70^) was used to determine significant taxa and EC numbers associated with co-variates, while adjusting for potential confounders. MaAslin2 was run on both genus- and species-level relative abundance data and EC number relative abundance data. All co-variates used in the ‘adonis’ analysis plus DOL were included as fixed effects in the analysis and subject ID was included as a random effect. The arcsin square root transformation was performed on relative abundance data and default MaAsLin2 parameters were used. All *P* values were adjusted by MaAsLin2 for multiple comparisons using FDR adjustment (Benjamini–Hochberg procedure) and the default *q*-value cut-off of 0.25 was used to identify significant results.

### Analysis of *B. infantis* HMO gene clusters

*B. infantis* HMO genes (as previously described^27^) were quantified by first identifying the corresponding UniRef90 gene families and then utilizing *B. longum*-stratified gene quantifications (quantifying UniRef90 gene families) from HUMAnN v.2 (ref. ^71^). Samples with >90% of the genes in these six genomic loci (H1, H2, H3, H4, H5 and a urease gene cluster) were classed as having *B. infantis*.

### Significance analysis of microarrays and metabolites, GO and enrichment analysis

Significance analysis of microarray (SAM) was used in MetaboAnalyst^67^ with a Delta threshold of 1.0 to identify specific metabolites discriminating PGCT-3 from PGCT-4/-5 and vice versa in both stool and serum.

### GO and enrichment analysis

GO and enrichment analysis were performed using the gprofiler2 package v.0.2.1 (ref. ^72^), with default parameters and a customized genetic background. The top 25 most significant GO biological processes for PGCT-4 and PGCT-5 were reported.

### Reporting summary

Further information on research design is available in the Nature Research Reporting Summary linked to this article.

### Supplementary information

Reporting summary

Supplementary Tables **Supplementary Table 1 Association between PGCT and clinical co-variates**. Global *P* values and adjusted odds ratios (AORs) with 95% CIs are based on the fitted mixed-effects logistic regression models, with patient ID as a random effect. **Supplementary Table** **2 MaAsLin2 results for significant taxa at the genus level**. Mixed-effects linear models using a variance-stabilizing arcsin square root transformation on relative abundance genera data were used to determine the significance. **Supplementary Table 3 MaAsLin2 results for significant taxa at the species level**. Mixed-effects linear models using a variance-stabilizing arcsin square root transformation on relative abundance species data were used to determine the significance. **Supplementary Table** **4 MaAsLin2 results for significant EC numbers**. Mixed-effects linear models using a variance-stabilizing arcsin square root transformation on relative abundance EC number data were used to determine the significance. **Supplementary Table 5 SAM results for significant metabolites in stool between PGCT-4/5 and PGCT-3. Supplementary Table 6 SAM results for significant metabolites in serum between PGCT-4/5 and PGCT-3. Supplementary Table 7 Top 30 most significantly enriched GO terms based on biological processes for PGCT-4 and PGCT-5**.

### Source data

Source Data Figs. 1–4 and Extended data Figs. 1–4. Statistical source data.

## Figures and Tables

**Extended Data Table 1:**
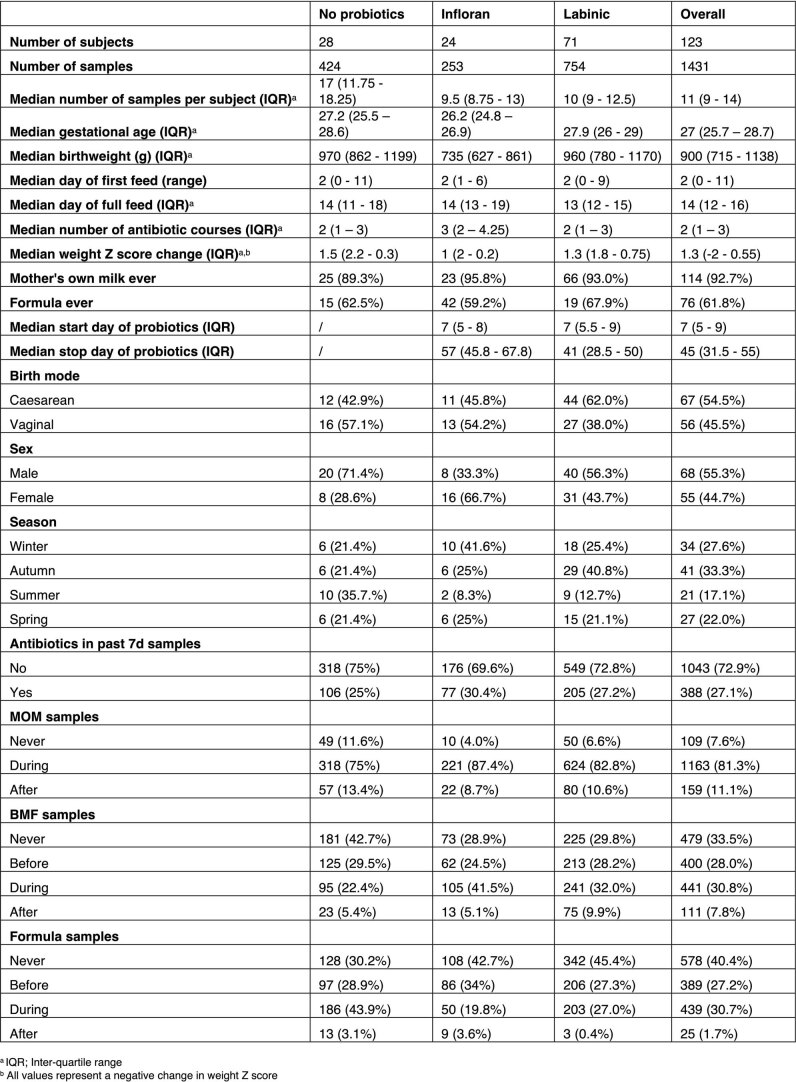
Demographics of the analytical cohort

**Extended Data Table 2:**
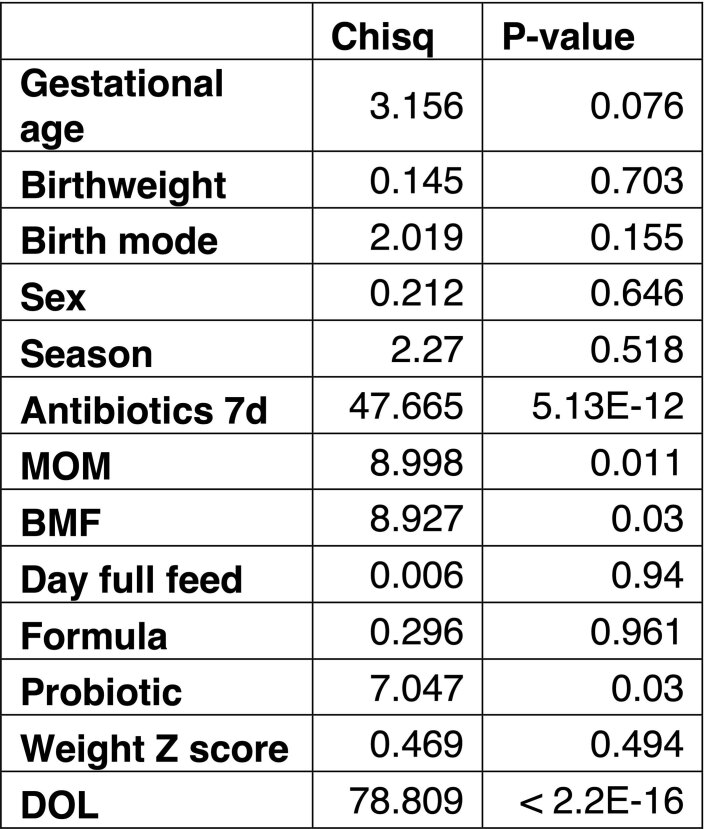
Association of clinical co-variates with Shannon diversity

Global *P* values and Wald’s χ^2^ test statistic for fixed effects are based on type II analysis of variance on the fitted linear mixed-effects model

**Extended Data Table 3:**
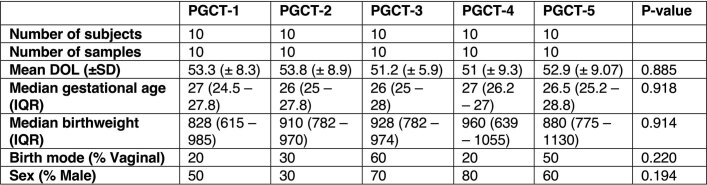
Clinical data for samples chosen for metabolomics and organoid experiments

*P* values are based on test for categorical data and Kruskal–Wallis test for continuous data

## Data Availability

Data are available in a public, open access repository. All metagenomic sequencing data generated and analysed in the present study have been deposited in the European Nucleotide Archive under study accession no. PRJEB49383. RNA-seq data generated and analysed in the present study have been deposited in the Sequencing Read Archive under study accession no. PRJNA859176. MS metabolomics data have been deposited in the EMBL-EBI MetaboLights database^73^ with the identifier MTBLS5406. Source data are provided with this paper.

## Extended data

are available for this paper at 10.1038/s41564-022-01213-w.

## Code availability

Source code for metabolomics data processing can be obtained at https://github.com/respiratory-immunology-lab/metabolome-lipidome-MSDIAL.
