# Supplementary material for: Strain-specific impacts of probiotics are a significant driver of gut microbiome development in very preterm infants
Source: Nat Microbiol. 2022 Sep 26;7(10):1525–35. doi: 10.1038/s41564-022-01213-w (PMC9519454; doi:10.1038/s41564-022-01213-w)
Supplement: Supplementary file 1 — Reporting summary [file 41564_2022_1213_MOESM1_ESM.pdf]

nature portfolio

Corresponding author(s): Janet E Berrington, Christoper J Stewart

Last updated by author(s): 15/07/2022

Reporting Summary

Nature Portfolio wishes to improve the reproducibility of the work that we publish. This form provides structure for consistency and transparency in reporting. For further information on Nature Portfolio policies, see our Editorial Policies and the Editorial Policy Checklist.

Statistics

For all statistical analyses, confirm that the following items are present in the figure legend, table legend, main text, or Methods section.

n/a	Confirmed	
☐	☒	The exact sample size (*n*) for each experimental group/condition, given as a discrete number and unit of measurement
☐	☒	A statement on whether measurements were taken from distinct samples or whether the same sample was measured repeatedly
☐	☒	The statistical test(s) used AND whether they are one- or two-sided<br>*Only common tests should be described solely by name; describe more complex techniques in the Methods section.*
☐	☒	A description of all covariates tested
☐	☒	A description of any assumptions or corrections, such as tests of normality and adjustment for multiple comparisons
☐	☒	A full description of the statistical parameters including central tendency (e.g. means) or other basic estimates (e.g. regression coefficient) AND variation (e.g. standard deviation) or associated estimates of uncertainty (e.g. confidence intervals)
☐	☒	For null hypothesis testing, the test statistic (e.g. *F*, *t*, *r*) with confidence intervals, effect sizes, degrees of freedom and *P* value noted<br>*Give P values as exact values whenever suitable.*
☒	☐	For Bayesian analysis, information on the choice of priors and Markov chain Monte Carlo settings
☐	☒	For hierarchical and complex designs, identification of the appropriate level for tests and full reporting of outcomes
☐	☒	Estimates of effect sizes (e.g. Cohen's *d*, Pearson's *r*), indicating how they were calculated

Our web collection on statistics for biologists contains articles on many of the points above.

Software and code

Policy information about availability of computer code

Data collection

No software were used for data collection.

Data analysis

For analyses carried out using R, V.4.3.1 was used in R Studio V4.0.2

Metagenomic data profiling:
Functional profiling: HUMAnN 2.0
Taxonomic profiling: MetaPhlAn 2.0
Viral profiling: VirMAP 1.0

Metabolomics data processing:
Sample processing: MS-DIAL V.4.8 (see code availability)
QC: R pmp V.1.6.0
Normalisation: R missForest package V.1.4

Transcriptomic data processing:
Processing: Illumina's bcl2fastq software
QC: FASTX Toolkit V.0.0.14
Read mapping: Salmon V.0.13.1
Normalise count data and find DEGs: R DESeq2 package V.1.32.0

Statistical analysis of all omic data:

Alpha diversity, 'adonis' PERMANOVA analysis and NMDS ordinations: R vegan package V.2.5-7
Visualisations: R ggplot package v.3.3.2
Linear and generalised linear mixed effects models: R glmmTMB package v.1.0.2.1
Logistic regression models when there was quasi complete or complete separation of data: R logistf package v.1.24 Diagnostic residual plots for models: R DHARMa package v.0.3.3.0
ANOVA: R car package V.3.0-10
Post-hoc tests: R emmeans package v.1.5.4 and
LEfSe: MicrobiomeAnalyst
MaAsLin2 v.1.2.0
Venn diagram: R VennDiagram package V.1.7.1
Mantel test: R ape package V.5.6-1
PLS-DA, PERMANOVA and SAM: MetaboAnalyst 5.0
GO and enrichment analysis: R gprofiler2 package V.0.2.1

Code availability:
Metabolomics processing: https://github.com/respiratory-immunology-lab/metabolome-lipidome-MSDIAL

For manuscripts utilizing custom algorithms or software that are central to the research but not yet described in published literature, software must be made available to editors and reviewers. We strongly encourage code deposition in a community repository (e.g. GitHub). See the Nature Portfolio guidelines for submitting code & software for further information.

Data

Policy information about availability of data

All manuscripts must include a data availability statement. This statement should provide the following information, where applicable:

- Accession codes, unique identifiers, or web links for publicly available datasets
- A description of any restrictions on data availability
- For clinical datasets or third party data, please ensure that the statement adheres to our policy

Data are available in a public, open access repository. All metagenomic sequencing data generated and analysed in this study have been deposited in the European Nucleotide Archive under study accession number PRJEB49383. RNA-sequencing data generated and analysed in this study have been deposited in the Sequencing Read Archive (SRA) under study accession number PRJNA859176. Mass spectrometry metabolomics data have been deposited to the EMBL-EBI MetaboLights database with the identifier MTBLS5406. Source data for all figures and extended data figures are provided with this paper.

Human research participants

Policy information about studies involving human research participants and Sex and Gender in Research.

Reporting on sex and gender	Not applicable.
Population characteristics	Detailed population characteristics are described in Extended data table 1.
Recruitment	Infants were recruited to the 'Supporting Enhanced Research in Vulnerable Infants Study' (SERVIS) with written parental consent covering data and sample collection. Infants born <32 weeks of gestation, who were not diagnosed with NEC, LOS, focal intestinal perforation, or other intestinal pathology, were included in the study.
Ethics oversight	The study protocol was approved by Newcastle Hospitals NHS Foundation Trust (NUTH), NRES Committee North East and N Tyneside 2 10/H0908/39, and the research complies with all relevant ethical regulations.

Note that full information on the approval of the study protocol must also be provided in the manuscript.

Field-specific reporting

Please select the one below that is the best fit for your research. If you are not sure, read the appropriate sections before making your selection.

☒ Life sciences ☐ Behavioural & social sciences ☐ Ecological, evolutionary & environmental sciences

For a reference copy of the document with all sections, see nature.com/documents/nr-reporting-summary-flat.pdf

Life sciences study design

All studies must disclose on these points even when the disclosure is negative.

Sample size	Sample size was determined by sample availability in the present observational study. The overall study analysed 1431 longitudinal stool samples from 123 infants, sufficient for a descriptive study of this nature. A subset of samples (10 for each PGCT, n = 50) were chosen for metabolomics which ensured statistical power. One organoid line was used, each experiment being performed in triplicate (one RNA sample failing QC), which we deemed sufficient for a pilot experiment of this nature.

Data exclusions	Some data was excluded from the study, this included samples taken before receipt of MOM (n = 6) because there was not sufficient power to draw any conclusions from these samples.
Replication	No replication was used on metagenomic data as this was an observational study. Experimental data (organoid model) were performed in three technical replicates.
Randomization	Randomization was not relevant to the current study due to the observational nature of the study. Covariates were controlled for in mixed models by including as fixed effects.
Blinding	No blinding was used as this was an observational study. For the organoid experiments, investigators were also not blinded.

Reporting for specific materials, systems and methods

We require information from authors about some types of materials, experimental systems and methods used in many studies. Here, indicate whether each material, system or method listed is relevant to your study. If you are not sure if a list item applies to your research, read the appropriate section before selecting a response.

Materials & experimental systems

n/a	Involved in the study
☒	☐ Antibodies
☐	☒ Eukaryotic cell lines
☒	☐ Palaeontology and archaeology
☒	☐ Animals and other organisms
☒	☐ Clinical data
☒	☐ Dual use research of concern

Methods

n/a	Involved in the study
☒	☐ ChIP-seq
☒	☐ Flow cytometry
☒	☐ MRI-based neuroimaging

Eukaryotic cell lines

Policy information about cell lines and Sex and Gender in Research

Cell line source(s)	his study used a preterm intestinal-derived organoid model which was established in the Stewart Lab, Newcastle University using resected surgical ileum tissue that was obtained from the neonatal intensive care unit of the Royal Victoria Infirmary, Newcastle. Informed consent was obtained by parents.
Authentication	The cell line was not authenticated.
Mycoplasma contamination	Cell lines were not tested for mycoplasma authentication.
Commonly misidentified lines (See ICLAC register)	No commonly misidentified cell lines were used.
